# Supplementary material for: Fast and exact gap-affine partial order alignment with POASTA
Source: Bioinformatics. 2025 Jan 3;41(1):btae757. doi: 10.1093/bioinformatics/btae757 (PMC11755094; doi:10.1093/bioinformatics/btae757)
Supplement: btae757_Supplementary_Data [file btae757_supplementary_data.zip › 034a0_Poasta_paper_OUP_Supplemental__Revision.pdf]

SUPPLEMENTAL MATERIALS

Fast and exact gap-affine partial order alignment with POASTA

Lucas R. van Dijk [1,2,*], Abigail L. Manson,[1] Ashlee M. Earl,[1] Kiran V. Garimella[3] and Thomas Abeel[1,2]

[1]Infectious Disease and Microbiome Program, Broad Institute of MIT and Harvard, 415 Main St, 02142, Cambridge, MA, USA, [2]Delft Bioinformatics Lab, TU Delft, Van Mourik Broekmanweg 6, 2628 XE, Delft, Zuid-Holland, The Netherlands and [3]Data Sciences Platform, Broad Institute of MIT and Harvard, 415 Main St, 02142, Cambridge, MA, USA

*Corresponding author. lvandijk@broadinstitute.org

Supplemental Figures 1

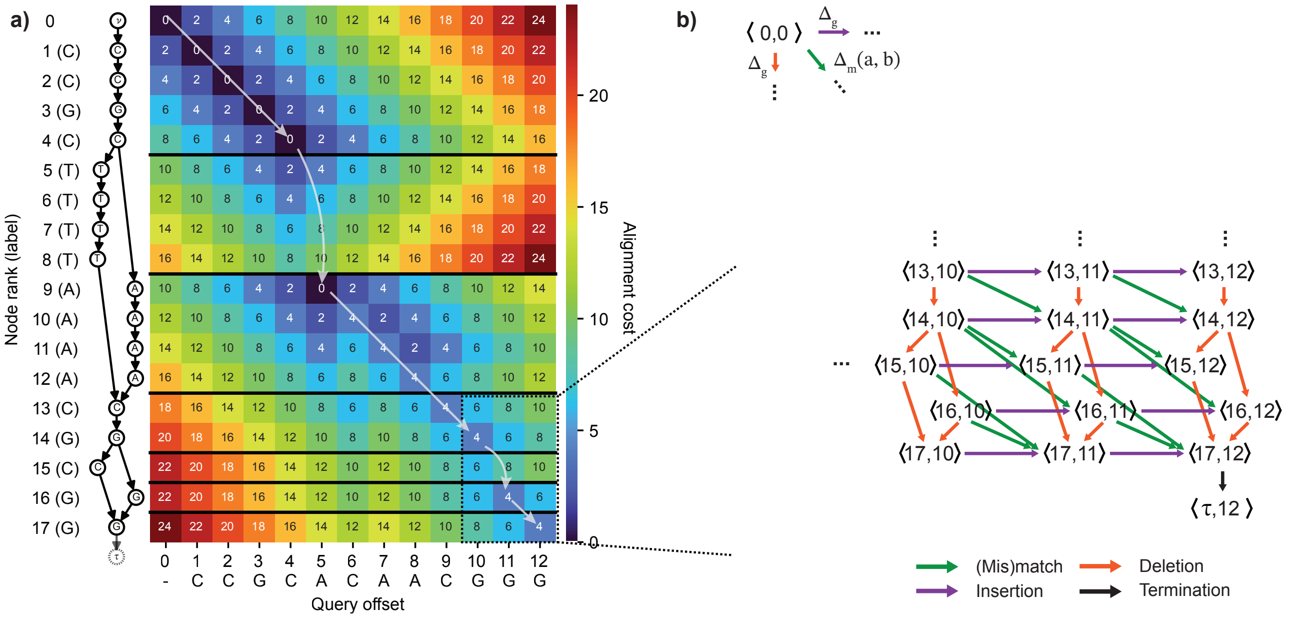

Fig. 1. **(a)** Example computation of aligning "CCGCACAACGGG" to a POA graph, with mismatch cost $\Delta_x = 4$, and gap cost $\Delta_g = 2$. The white arrows indicate the optimal alignment path. **(b)** A subgraph of the full *alignment graph*, corresponding to POA graph nodes 13-17, and query offset 10-12. A node $\langle v, o \rangle$ in the alignment graph represents a cursor to a node in the POA graph v and a query offset o. The various alignment operations ((mis)match, insertion, deletion) correspond to different kinds of edges. Insertion and deletion edges are weighted with the gap cost Δ_g, and (mis)match edges with a function $\Delta_m(a, b) = \{\Delta_x$ if $a \neq b$, and 0 otherwise.

© The Author 2024. Published by Oxford University Press. All rights reserved. For permissions, please e-mail: journals.permissions@oup.com

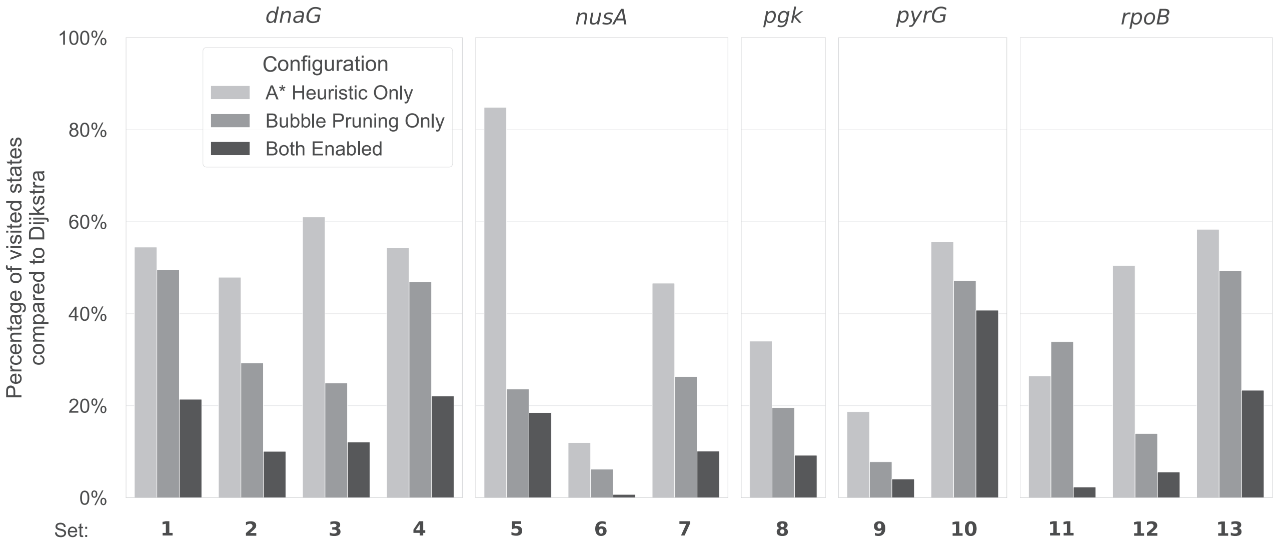

Set:	1	2	3	4	5	6	7	8	9	10	11	12	13
# genes:	141	422	338	319	373	577	159	1173	179	315	1038	425	2385
Length:	1767	1760	1746	1680	1011	1482	1488	1163	1601	1617	4065	4029	4020
± std:	±15	±73	±3	±3	±28	±3	±3	±22	±87	±0	±137	±1	±164
ANI:	83%	88%	93%	96%	87%	91%	93%	91%	95%	97%	90%	90%	92%

Fig. 2. POASTA's A* heuristic and superbubble-informed pruning substantially reduces the number of visited alignment states. Barplot indicating the percentage of visited alignment states of three POASTA configurations compared to a Dijkstra baseline (i.e., with both the A* heuristic and bubble pruning disabled).

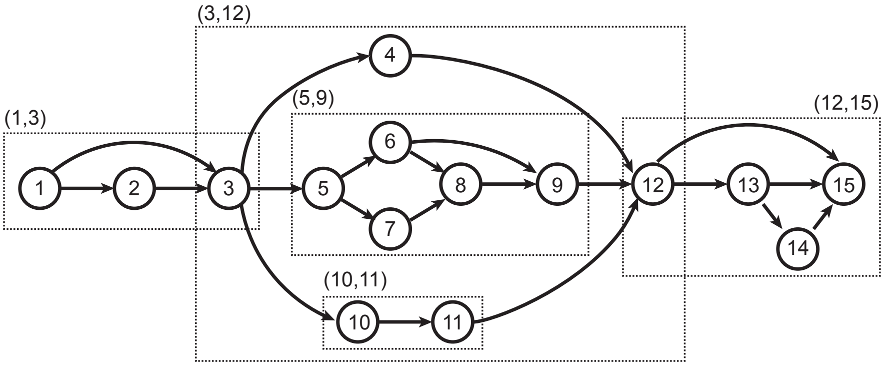

Fig. 3. An example graph containing multiple superbubbles. Each superbubble is marked with a dotted rectangle, labeled with its (*entrance*, *exit*). Superbubbles can be nested within each other: superbubbles (5, 9) and (10, 11) are contained within superbubble (3, 12). Superbubble (10, 11) is an example of a superbubble without an interior.

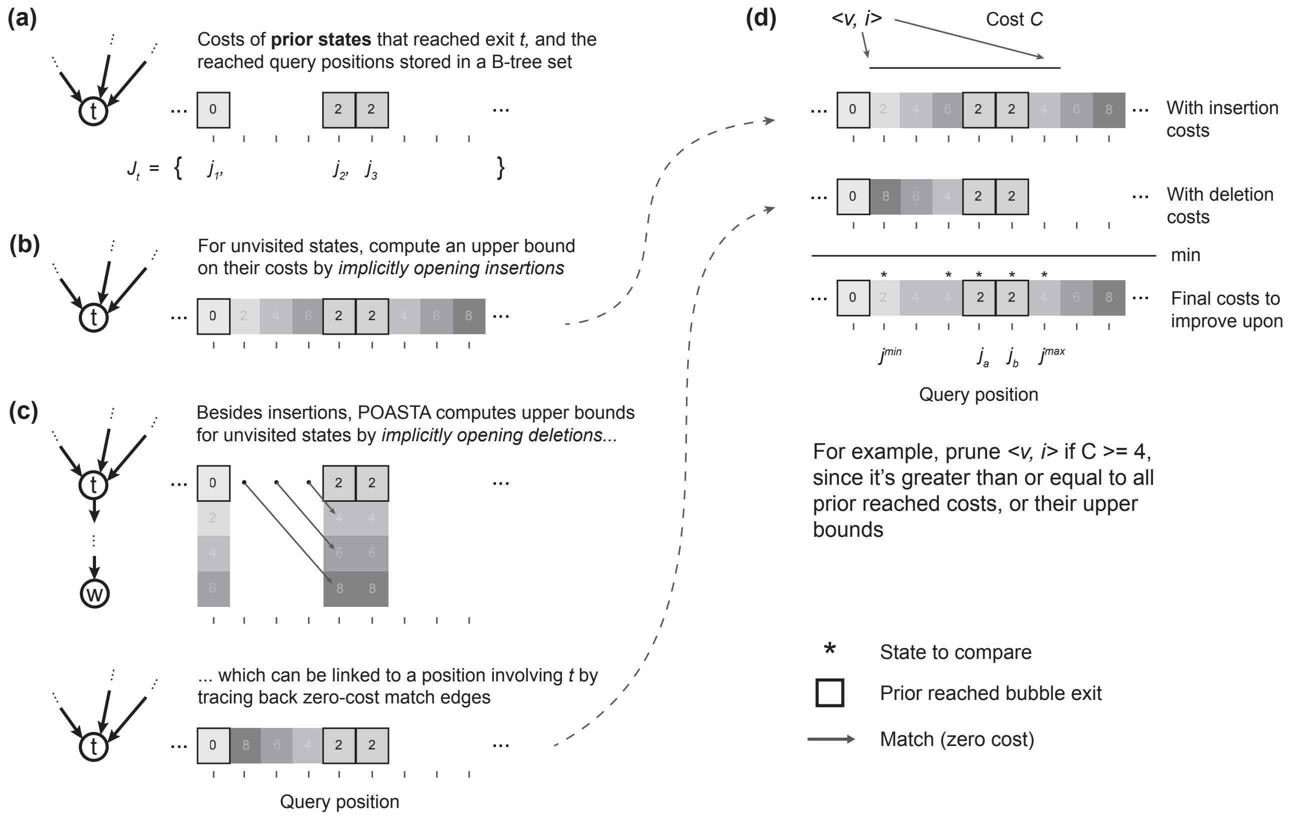

Fig. 4. POASTA considers all prior reached bubble exits when testing if a state can be pruned. **(a)** Example that shows alignment states that reached exit t previously at query position j_1, j_2 and j_3 (bordered squares). Positions are stored as an ordered set J_t. **(b)** Example upper bounds for unvisited states by implicitly opening insertions (squares without border). **(c)** Example upper bounds for unvisited states by implicitly opening deletions (top; squares without border), reaching some node w downstream of t. Tracing back zero-cost match edges (black arrows) from opened deletions enables linking the upper bound to a query position for t (bottom). **(d)** Example of how POASTA determines whether to prune a state $\langle v, i \rangle$ reached with alignment cost C. POASTA determines the lowest upper bound from implicitly opened gaps for each query position by taking the minimum cost of an implicitly opened insertion or deletion. POASTA only needs to compare the alignment cost C with the upper bounds for states marked with a *. All examples use the gap-linear cost model with $\Delta_g = 2$.

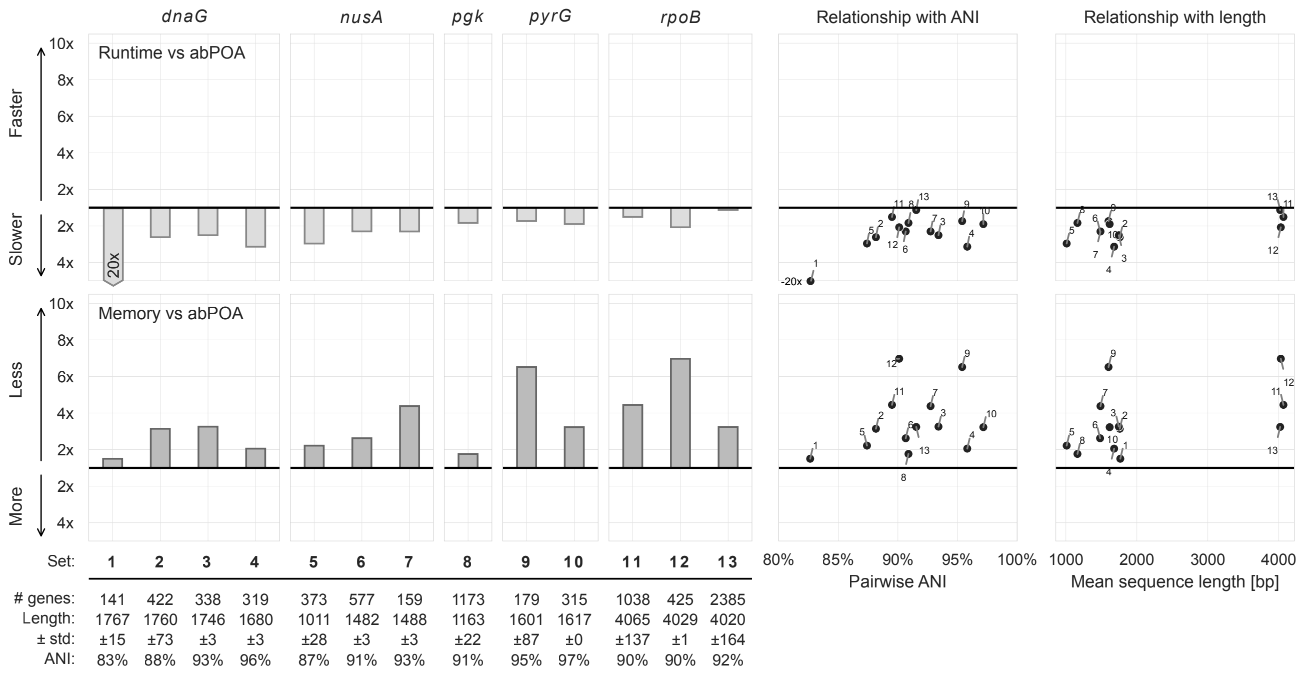

Fig. 5. abPOA is, on average, 3.5x faster than POASTA, but doesn't guarantee optimal alignment and uses more memory. **(a)** Relative runtime of POASTA compared to abPOA for each set of gene sequences. **(b)** The relationship between pairwise ANI of each gene sequence set and POASTA's relative runtime. **(c)** The relationship between mean sequence length and POASTA's relative runtime. **(d)** Relative memory usage of POASTA compared to abPOA for each set of gene sequences. **(e)** The relationship between pairwise ANI of each sequence set and POASTA's relative memory usage. **(f)** The relationship between the mean sequence length of each sequence set and POASTA's relative memory usage.

Supplemental Methods

The connection between dynamic programming recurrence and the alignment graph

Gap-linear alignment costs

The conventional dynamic programming (DP) recurrence for POA with gap-linear costs is (Lee et al., 2002):

$$S_{v,i} = \min \begin{cases} S_{u,i-1} + \Delta(\sigma(v), q_i) & \forall u : (u,v) \in E \\ S_{u,i} + \Delta_g & \forall u : (u,v) \in E \\ S_{v,i-1} + \Delta_g \end{cases} \quad (1)$$

Here, $\sigma(v) \to \Sigma$ returns the node label for a node v, and $\Delta(a,b) \to \mathbb{Z}$ is a function that returns the match cost Δ_m if $a = b$, and mismatch cost Δ_x if $a \neq b$. The three cases correspond to a (mis)match between the graph and query, opening or extending a deletion, and opening or extending an insertion.

To translate the recurrence to edges in an alignment graph, we define the edge set E^A as follows. Edges connect two alignment states $\langle u, i\rangle \to \langle v, j\rangle$ if one of the following conditions hold:

- **Match and mismatch.** $(u,v) \in E$, $v \neq \tau$, and $i + 1 = j$, $j \leq m$, with the (mis)match cost $\Delta(\sigma(v), q_j)$ as weight;
- **Deletion.** $(u,v) \in E$, $v \neq \tau$, and $i = j$, with the gap cost Δ_g as weight;
- **Insertion.** $u = v$, $u, v \neq \tau$, and $i + 1 = j$, $j \leq m$, with the gap cost Δ_g as weight;
- **Termination.** $(u,v) \in E$, $v = \tau$, and $i = j = m$, with zero cost.

These edges (except for the termination edge) are analogous to the different cases in Equation 1. We note that edges originating from the special start node ν are analogous to the base cases in the dynamic programming problem, i.e., the first row and column of the matrix initialized with the gap costs. Edges towards to special termination node τ have no analogous case in the dynamic programming recurrence and therefore have zero cost.

Gap-affine alignment costs

To compute the gap-affine alignment, the Smith-Waterman-Gotoh (SWG) DP recurrence for affine pairwise alignment (Gotoh, 1982) can be adapted to POA as follows:

$$\begin{cases} I_{v,i} & = \min\{M_{v,i-1} + \Delta_o + \Delta_e,\ I_{v,i-1} + \Delta_e\} \\ D_{v,i} & = \min\{M_{u,i} + \Delta_o + \Delta_e,\ D_{u,i} + \Delta_e\} \\ & \qquad \forall u : (u,v) \in E \\ M_{v,i} & = \min\{I_{v,i},\ D_{v,i},\ M_{u,i-1} + \Delta(\sigma(v), q_i)\} \\ & \qquad \forall u : (u,v) \in E \end{cases} \quad (2)$$

The two cases for $I_{v,i}$ correspond to opening an insertion and extending an insertion; the two cases for $D_{v,i}$ correspond to opening a deletion and extending a deletion; and the three cases for $M_{v,i}$ correspond to closing an insertion, deletion, or a (mis)match.

To extend the alignment graph formulation to the gap-affine model, with gap open cost Δ_o and gap extend cost Δ_e, we define the node set of the gap-affine alignment graph as follows: $V^A = (V \times \{0, \ldots, m\} \times \{M, D, I\})$. In other words, for each pair $v \in V, i \in [0, m]$, we now have three possible alignment states: $\langle v, i, M\rangle$, $\langle v, i, D\rangle$, $\langle v, i, I\rangle$, representing the match, deletion, and insertion state, respectively. Edges in the gap-affine alignment graph are defined as follows:

- **Edges ending in the insertion state**
  - $\langle u, i, M\rangle \to \langle u, i+1, I\rangle$, $u \neq \tau$, $i+1 \leq m$, weighted with gap open cost $\Delta_o + \Delta_e$
  - $\langle u, i, I\rangle \to \langle u, i+1, I\rangle$, $u \neq \tau$, $i+1 \leq m$, weighted with gap extend cost Δ_e
- **Edges ending in the deletion state**
  - $\langle u, i, M\rangle \to \langle v, i, D\rangle$, $(u,v) \in E$, $v \neq \tau$, weighted with gap open cost $\Delta_o + \Delta_e$
  - $\langle u, i, D\rangle \to \langle v, i, D\rangle$, $(u,v) \in E$, $v \neq \tau$, weighted with gap extend cost Δ_e
- **Edges ending in the (mis)match state**
  - $\langle u, i, M\rangle \to \langle v, i+1, M\rangle$, $(u,v) \in E$, $v \neq \tau$, $i+1 \leq m$, with (mis)match cost $\Delta(\sigma(v), q_{i+1})$
  - $\langle u, i, I\rangle \to \langle u, i, M\rangle$, $u \neq \tau$, weighted with zero cost
  - $\langle u, i, D\rangle \to \langle u, i, M\rangle$, $u \neq \tau$, weighted with zero cost
- **Termination edges**
  - $\langle u, m, M\rangle \to \langle \tau, m, M\rangle$, $(u, \tau) \in E$, weighted with zero cost

These edges are analogous to the cases in Equation 2.

Proof of minimum number of indel edges

Given an alignment state $\langle u, i\rangle$, let $d^{\min}_{u,\tau}$ and $d^{\max}_{u,\tau}$ be the minimum and maximum path length in the POA graph from u to end node τ. We additionally compute the length of the unaligned query sequence $l_r = m - i$. The minimum number of indel edges to traverse is then:

Definition 1 (Minimum number of indel edges)

$$N^{\min}_g = \begin{cases} l_r - (d^{\max}_{u,\tau} - 1) & \text{if } d^{\max}_{u,\tau} - 1 < l_r \\ (d^{\min}_{u,\tau} - 1) - l_r & \text{if } d^{\min}_{u,\tau} - 1 > l_r \\ 0 & \text{otherwise} \end{cases} \quad (3)$$

We subtract one from $d^{\min}_{u,\tau}$ and $d^{\max}_{u,\tau}$ to exclude the edge towards τ.

Proof Let $\mathcal{W} \subset V$ be the subset of POA graph nodes with an outgoing edge to τ, i.e., $\mathcal{W} = \{w : (w, \tau) \in E\}$. By definition of the alignment graph, the alignment termination state is only reachable from alignment states $\langle w, m\rangle : w \in \mathcal{U}$. We will prove each case separately.

In the first case, $d^{\max}_{u,\tau} - 1 < l_r$. By definition of $\mathcal{W}$, $\exists w \in \mathcal{W}$ such that $d_{u,w} = d^{\max}_{u,\tau} - 1$, i.e., excluding the last edge towards τ from the maximum path length. The presence of this maximum length path in the POA graph implies a corresponding path of all (mis)match edges in the alignment graph, reaching the alignment state $\langle w, j\rangle, w \in \mathcal{W}, j = i + d_{v,w}$. Since this traversed the maximum length path in the POA graph, j is also the maximum query position reachable from $\langle u, i\rangle$. Since $d_{u,w} < l_r$, we infer that $j < m$. This means that the alignment termination state is not reachable from $\langle u, j\rangle$,

and at least $m - j = l_r - (d^{\max}_{v,\tau} - 1)$ insertion edges need to be traversed to be able to reach the alignment termination state.

In the second case, $d^{\min}_{v,\tau} - 1 > l_r$. Similarly as above, $\exists w \in \mathcal{W}$, such that $d_{u,w} = d^{\min}_{u,\tau} - 1$, i.e., excluding the last edge towards τ from the minimum path length. To reach the alignment termination state from $\langle u, i\rangle$, we need to traverse at least $d_{u,w}$ (mis)match or deletion edges, since this is the minimum length path to the POA end node. We can, however, traverse only l_r (mis)match edges, since no (mis)match edges exist that would move the query position beyond the query sequence length m. After traversing l_r (mis)match edges, we would reach some state $\langle v, m\rangle$, with v being a node on the minimum path in the POA graph $u \to \ldots \to v \to \ldots \to w \to \tau$. To be able to reach the alignment termination state, we need to traverse at least $d^{\min}_{v,\tau} - 1 - l_r$ deletion edges.

In the last case, $d^{\min}_{v,\tau} - 1 < l_r < d^{\max}_{v,\tau} - 1$, which implies that there exist a path from $\langle u, i\rangle$ to $\langle \tau, m\rangle$ without the need to traverse any indel edges. □

Extension of the minimum gap cost heuristic function to the gap-affine model

To compute the minimum gap cost heuristic using gap-affine model, we need to take into account that insertion or deletion states do not need to incur the gap-open cost again.

A state $\langle v, i, M\rangle$ always needs to incur the gap-open cost, thus the heuristic is computed as follows:

Definition 2 $$h\langle v,i,M\rangle = \begin{cases} 0 & \text{if } N^{\min}_g = 0 \\ \Delta_o + N^{\min}_g \Delta_e & \text{otherwise} \end{cases}$$

A state $\langle v, i, I\rangle$ is already in insertion state and would not have to incur the gap open cost again if $d^{\max}_{v,\tau} - 1 < l_r$, since the minimum number of indel edges (as described above) are all insertion edges in that case. We compute the heuristic as follows:

Definition 3 $$h\langle v,i,I\rangle = \begin{cases} 0 & \text{if } N^{\min}_g = 0 \\ N^{\min}_g \Delta_e & \text{if } d^{\max}_{v,\tau} - 1 < l_r \\ \Delta_o + N^{\min}_g \Delta_e & \text{otherwise} \end{cases}$$

Similarly, for a state $\langle v, i, D\rangle$, we would not have to incur the gap open cost again if $d^{\min}_{v,\tau} - 1 > l_r$, since the minimum number of indel edges are all deletion edges in that case. The heuristic is computed as follows:

Definition 4 $$h\langle v,i,D\rangle = \begin{cases} 0 & \text{if } N^{\min}_g = 0 \\ N^{\min}_g \Delta_e & \text{if } d^{\min}_{v,\tau} - 1 > l_r \\ \Delta_o + N^{\min}_g \Delta_e & \text{otherwise} \end{cases}$$

Implementation details of superbubble-informed pruning

Effective detection of prunable states by computing implicitly opened gap costs

To test if a state $\langle v, i\rangle$ reached at cost C and contained in a superbubble (s, t) can be pruned, POASTA infers the range of states $\langle t, j^{\min}\rangle, \ldots, \langle t, j^{\max}\rangle$ reachable with zero-cost match edges (Methods; Main Text Figure 3c). A naive approach would scan the entire range $\langle t, j^{\min}\rangle, \ldots, \langle t, j^{\max}\rangle$ and assess whether all of those states were visited prior at a lower or equal cost to C. This would be ineffective for two reasons: 1) for larger and more complex bubbles, the range $j^{\min}, \ldots, j^{\max}$ can be quite large, and 2) at the time of testing, many of those states might not have been reached yet, thus without a known alignment cost to compare to C.

To more effectively detect prunable states, POASTA employs the following: First, POASTA tracks in a B-tree set on which query positions a superbubble exit t have been reached (Supplementary Figure 4a). Then, POASTA uses the inherent ordering in a B-tree to quickly retrieve which query positions have reached bubble exit t in the range $j^{\min}, \ldots, j^{\max}$. Finally, POASTA computes upper bounds on the alignment costs for unvisited positions in this range by *implicitly opening gaps* from visited positions (Supplementary Figure 4bc). A state $\langle v, i\rangle$ will be pruned if its alignment cost C is greater than or equal to the (upper bound on) costs for states $\langle t, j^{\min}\rangle, \ldots, \langle t, j^{\max}\rangle$ (Supplementary Figure 4d). We will detail each step below.

First, the B-tree for each superbubble exit is an ordered set $J_t = \{j_1, j_2, \ldots, j_n\}$ of query positions on which a superbubble exit has been reached. Each time a state $\langle t, j\rangle$ is popped from the A* queue, POASTA inserts j into the B-tree for an exit t (Supplementary Figure 4a).

Second, as discussed above, POASTA assesses whether a state $\langle v, i\rangle$ can be pruned by comparing its alignment cost C to the alignment costs of $\langle t, j^{\min}\rangle, \ldots, \langle t, j^{\max}\rangle$. Using the inherent ordering in the B-tree, POASTA can quickly find indices a and b using binary search such that $j^{\min} \leq j_a, \ldots, j_b \leq j^{\max}$, i.e., the list of visited query positions in the range $[j^{\min}, j^{\max}]$.

POASTA uses this list of visited query positions to compute upper bounds on the alignment cost for the *unvisited* query positions, i.e., states $\langle t, j'\rangle : j' \in [j^{\min}, j^{\max}], j' \notin J_t$, by *implicitly opening gaps*. We call this implicitly opening gaps since these upper bounds are computed on the fly, not recorded anywhere, and not included in the A* queue.

For example, if $\langle t, j\rangle$ was previously visited with a cost $C_{\langle t,j\rangle}$, then any unvisited state $\langle t, j'\rangle : j' > j$ could also be reached by opening an insertion from $\langle t, j\rangle$. In the case of linear gap penalties, these states would then be reached at an alignment cost $C_{\langle t,j'\rangle} = C_{\langle t,j\rangle} + \Delta_g(j' - j)$. This is an upper bound on the cost for a state $\langle t, j'\rangle$, since there may exist a path to that state with a lower alignment cost (Supplementary Figure 4b).

Besides opening an insertion, we could also open a deletion from a previously reached state $\langle t, j\rangle$, reaching some state $\langle w, j\rangle$ where w is a node downstream of t. In the case of linear gap penalties, this state would be reached with an alignment cost of $C_{\langle w,j\rangle} = C_j + \Delta_g d_{t,w}$, where $d_{t,w}$ is the path length between t and w. This is again an upper bound on the cost for state $\langle w, j\rangle$, since there may be other paths with lower alignment costs. We link the upper bound of $\langle w, j\rangle$ to a state involving exit t and a query position $j'' < j$ by noting that any alignment path from a state $\langle v, i\rangle$ to $\langle w, j\rangle$ would need to traverse an alignment state $\langle t, j''\rangle : i \leq j'' \leq j$ since v is part of a superbubble with exit t. Tracing back the best-case scenario of zero-cost match edges from $\langle w, j\rangle$, we find that $j'' = j - d_{t,w}$. Thus, for $\langle v, i\rangle$ to improve over the upper bound for $\langle w, j\rangle$, it would also need to reach $\langle t, j - d_{t,w}\rangle$ with an alignment cost lower than $C_{\langle w,j\rangle}$ (Supplementary Figure 4c).

Finally, while implicitly opening gaps enables computing upper bounds of the alignment cost for any state in the range $\langle t, j^{\min}\rangle, \ldots, \langle t, j^{\max}\rangle$, POASTA only needs to check a subset of positions in this range when assessing to prune a state $\langle v, i\rangle$. Specifically, POASTA exploits the fact that the cost of a gap linearly increases with its length. For example, if an exit t has

been reached on query positions $j_1, j_2 \in J_T, j_2 \gg j_1$, the position with the *lowest* implicit insertion cost in the range $[j_1, j_2]$ would be $j_1 + 1$, since for all following positions, the insertion cost would only increase. Similarly, the position with the lowest implicit deletion cost in the range would be $j_2 - 1$. By comparing the (upper bound on) alignment costs for the subset of positions $\{j^{\min}, j^{\max}\} \cup \{j-1, j, j+1 : j \in J_t^{[a,b]}\}$ to the alignment cost C of a state $\langle v, i \rangle$, POASTA thus determines whether $\langle v, i \rangle$ can improve the alignment score over the entire range $\langle t, j^{\min} \rangle, \ldots, \langle t, j^{\max} \rangle$ (Supplementary Figure 4d).

Detecting prunable states with gap-affine penalties

Superbubble-informed pruning is straightforward to adapt to the gap-affine cost model. One option would be to keep separate, ordered sets of reached positions J_t^M, J_t^D, J_t^I for matches, deletions, and insertions. When testing to prune a state $\langle v, i, M \rangle$, we could open gaps from positions in J_t^M, while considering the additional gap open cost. When testing to prune a state $\langle v, i, I \rangle$ or $\langle v, i, D \rangle$, we could extend gaps from positions in J_t^I and J_t^D, respectively, without incurring the gap open cost. However, the downside of such an approach is the additional cost of inserting an increased number of positions into a B-tree, which has logarithmic time complexity.

Instead, POASTA employs another option: it tracks only reached (mis)match states in J_t^M, thus substantially reducing the number of times it needs to insert a position in a B-tree. POASTA can still use the positions in J_t^M to compute implicit gap costs and test whether to prune states $\langle v, i, I \rangle$ or $\langle v, i, D \rangle$. One thing to consider is that the latter states will not need to incur the gap open cost for extending the insertion or the deletion, while implicit gaps from positions in J_t^M do. Thus, POASTA will not prune a state $\langle v, i, I \rangle$ or $\langle v, i, D \rangle$, reached at cost C, if $\exists j \in J_t^M : C < C_{\langle t,j \rangle} + \Delta_o$.

Construction of benchmark datasets

To construct our bacterial gene benchmark datasets, we first downloaded all bacterial "complete" genomes from NCBI RefSeq (40,188 genomes total; accessed July 2023). We used the accompanying gene annotations to extract the *dnaG*, *nusA*, *pgk*, *pyrG*, and *rpoB* gene sequences from each genome.

To create each individual benchmark set, we clustered gene sequences using single-linkage hierarchical clustering, as implemented in SciPy (Virtanen et al., 2020). Pairwise genetic distances were estimated using Mash (Ondov et al. (2016); $k = 15$; sketch size = 5,000), and were additionally used to deduplicate the sequence set, selecting one representative per set of identical sequences. We set the clustering threshold to 0.1, i.e., a new cluster would be formed if no neighbor could be found with a genetic distance < 0.1. This threshold is coarse enough to generate multiple genus and species-level clusters. We picked one or more clusters for each gene family as final datasets, each with at least 100 sequences, and varying the pairwise average nucleotide identities (ANI). Finally, each set was sorted by picking one "center" sequence with the smallest average Mash distance to all others and then ordering the remaining sequences in the set by the distance to the chosen "center" sequence, a strategy commonly applied before POA Gao et al. (2021).

Benchmark execution details

We ran POASTA with the following parameters: mismatch cost $\Delta_x = 4$, gap open cost $\Delta_o = 6$, and gap extend cost $\Delta_e = 2$, the same costs as used in the Wavefront Algorithm (WFA) (Marco-Sola et al., 2021). Our benchmark suite calls POASTA's Rust API directly to perform alignments. Thus, its runtime and memory usage measurements exclude anything related to startup or file input/output.

We wrote Rust bindings to SPOA and abPOA to achieve the same for those tools. All tools were configured to perform global alignment using the same cost model. Tools were run in single-threaded mode on a `c2-standard-8` virtual machine on the Google Cloud Platform, with an Intel Cascade Lake CPU and with 32 GB of RAM.

Assessing the frequency of missed optimal alignments with abPOA

To assess how frequently abPOA missed the optimal alignment, we constructed a graph comprising ten randomly selected gene sequences for each benchmark set. The remaining sequences were then aligned to the graph without updating it. This ensured each alignment was performed against the same graph and alignment scores were not influenced by different alignment backtracking or graph update choices. We performed alignments with SPOA, POASTA, and abPOA (each with the same graph as input) and recorded the alignment score for each non-graph sequence alignment.

We identified a discrepancy in abPOA's graph implementation, which allowed alignments to start at any node that is the start of a previously added sequence to the graph. This enables the alignment to potentially skip nodes, whereas SPOA/POASTA would incur additional indel costs. It similarly allowed alignments to end at any node representing the end of a sequence added to the graph. This discrepancy resulted in better (i.e., lower) alignment costs than expected since we benchmarked global alignment where indels at the start or end still incur an alignment cost.

Construction of **Mycobacterium tuberculosis** dataset

To construct the benchmark sets with *Mycobacterium tuberculosis* genomic sequences of 250, 500, and 1000 kbp in length, we downloaded all "complete" *M. tuberculosis* genomes available on NCBI RefSeq (370 total; accessed November 2023). To make all genomes colinear, we rotated and reoriented each genome such that each started with the gene *dnaA*, using the `fix-start` utility in Circlator (Hunt et al., 2015). Additionally, since inversions also break co-linearity, and POA poorly supports aligning large inversions, we excluded 29 genomes with more than 15% ($\geq$ 660 kbp) of its genome inverted with respect to the canonical reference *M. tuberculosis* H37Rv, detected using MUMMER (Marçais et al., 2018).

We truncated genomes at specific genes to obtain sequences of the desired length. For the 250 kbp, 500 kbp, and 1 Mbp datasets, we used the genes *trmB*, *thiE*, and *gltA2* as cutoff points, respectively. We manually confirmed that these genes were located around the 250 kbp, 500 kbp, and 1 Mbp marks in each of the *dnaA* rotated and reoriented genomes. Finally, each dataset was sorted such that references were in ascending order of their Mash distance (Ondov et al., 2016) to H37Rv.

POASTA was executed with the same alignment cost model as described above, but on the larger `c2-standard-60` virtual machine on the Google Cloud Platform, which has 240 GB of RAM available.

References

Y. Gao, Y. Liu, Y. Ma, B. Liu, Y. Wang, and Y. Xing. abPOA: an SIMD-based C library for fast partial order alignment using adaptive band. *Bioinformatics*, 37(15):2209–2211, Aug. 2021. ISSN 1367-4803. doi: 10.1093/bioinformatics/btaa963. URL https://doi.org/10.1093/bioinformatics/btaa963.

O. Gotoh. An improved algorithm for matching biological sequences. *Journal of Molecular Biology*, 162(3):705–708, Dec. 1982. ISSN 0022-2836. doi: 10.1016/0022-2836(82)90398-9. URL https://www.sciencedirect.com/science/article/pii/0022283682903989.

M. Hunt, N. D. Silva, T. D. Otto, J. Parkhill, J. A. Keane, and S. R. Harris. Circlator: automated circularization of genome assemblies using long sequencing reads. *Genome Biology*, 16(1):294, Dec. 2015. ISSN 1474-760X. doi: 10.1186/s13059-015-0849-0. URL https://doi.org/10.1186/s13059-015-0849-0.

C. Lee, C. Grasso, and M. F. Sharlow. Multiple sequence alignment using partial order graphs. *Bioinformatics*, 18(3):452–464, Mar. 2002. ISSN 1367-4803. doi: 10.1093/bioinformatics/18.3.452. URL https://doi.org/10.1093/bioinformatics/18.3.452.

S. Marco-Sola, J. C. Moure, M. Moreto, and A. Espinosa. Fast gap-affine pairwise alignment using the wavefront algorithm. *Bioinformatics*, 37(4):456–463, Feb. 2021. ISSN 1367-4803. doi: 10.1093/bioinformatics/btaa777. URL https://doi.org/10.1093/bioinformatics/btaa777.

G. Marçais, A. L. Delcher, A. M. Phillippy, R. Coston, S. L. Salzberg, and A. Zimin. MUMmer4: A fast and versatile genome alignment system. *PLoS Comput. Biol.*, 14(1):e1005944, Jan. 2018. ISSN 1553-734X. doi: 10.1371/journal.pcbi.1005944. URL http://dx.doi.org/10.1371/journal.pcbi.1005944.

B. D. Ondov, T. J. Treangen, P. Melsted, A. B. Mallonee, N. H. Bergman, S. Koren, and A. M. Phillippy. Mash: fast genome and metagenome distance estimation using MinHash. *Genome Biol.*, 17(1):132, June 2016. ISSN 1465-6906. doi: 10.1186/s13059-016-0997-x. URL http://dx.doi.org/10.1186/s13059-016-0997-x.

P. Virtanen, R. Gommers, T. E. Oliphant, M. Haberland, T. Reddy, D. Cournapeau, E. Burovski, P. Peterson, W. Weckesser, J. Bright, S. J. van der Walt, M. Brett, J. Wilson, K. J. Millman, N. Mayorov, A. R. J. Nelson, E. Jones, R. Kern, E. Larson, C. J. Carey, I. Polat, Y. Feng, E. W. Moore, J. VanderPlas, D. Laxalde, J. Perktold, R. Cimrman, I. Henriksen, E. A. Quintero, C. R. Harris, A. M. Archibald, A. H. Ribeiro, F. Pedregosa, and P. van Mulbregt. SciPy 1.0: fundamental algorithms for scientific computing in Python. *Nature Methods*, 17(3):261–272, Mar. 2020. ISSN 1548-7105. doi: 10.1038/s41592-019-0686-2. URL https://www.nature.com/articles/s41592-019-0686-2. Number: 3 Publisher: Nature Publishing Group.
